# Ultrasonographic findings and prenatal diagnosis of complete trisomy 17p syndrome: A case report and review of the literature

**DOI:** 10.1002/jcla.23582

**Published:** 2020-09-20

**Authors:** Linlin Li, Xinyue Zhang, Qingyang Shi, Leilei Li, Yuting Jiang, Ruizhi Liu, Hongguo Zhang

**Affiliations:** ^1^ Center for Reproductive Medicine and Center for Prenatal Diagnosis First Hospital Jilin University Changchun China

**Keywords:** balanced translocation, complete trisomy 17p syndrome, prenatal diagnosis, SNP array, ultrasonographic finding

## Abstract

**Background:**

Trisomy of the short arm of chromosome 17 is a rare genomic disorder. The clinical features of complete trisomy 17p syndrome have been described. Most cases of this syndrome have been found in infants and children, but only a few cases were found by ultrasound in the prenatal period.

**Methods:**

We report a case of complete trisomy 17p syndrome, which was inherited from paternal balanced translocation t(15;17)(q11.2;q11.2). A pregnant woman underwent an ultrasound examination at 24 weeks of gestation. Amniotic fluid was collected by amniocentesis. Cytogenetic and single nucleotide polymorphism array analyses were performed. We further reviewed the relationship between duplication regions and the clinical phenotype.

**Results:**

Ultrasonographic evaluation showed intrauterine growth retardation and a right choroid plexus cyst, but the gallbladder was not observed. The fetal karyotype was 46,XX,der(17)t(15;17)(q11.2;q11.2)pat. The father's karyotype was 46,XY,t(15;17)(q11.2;q11.2). The single nucleotide polymorphism array results showed arr[GRCh37] 17p13.3q11.1(525‐25309337)×3, which indicated a 25.309‐Mb duplication.

**Conclusion:**

Complete trisomy 17p syndrome shows severe malformations. Intrauterine growth retardation is the most typical manifestation of this syndrome as shown by ultrasonography in the second trimester of pregnancy. The genotype‐phenotype relationships of complete trisomy 17p syndrome are not completely consistent. To further determine these relationships, additional cases are necessary to provide more information from ultrasonographic findings during pregnancy.

## 1 INTRODUCTION

Trisomy of the short arm of chromosome 17 is a rare genomic disorder.^1^ The clinical features of complete trisomy 17p syndrome have been previously described.^2^, ^3^, ^4^ These mainly include prenatal and postnatal growth retardation, craniofacial anomalies, short webbed neck, hypotonia, cardiac malformations, joint contractures, abnormality of the iris, and varying degrees of hearing loss. Most carriers have been reported in infants and childhood, but only a few cases have been described by ultrasound in the prenatal period.^1^, ^2^, ^5^, ^6^, ^7^, ^8^ Therefore, further studies are required to determine the genotype‐phenotype correlation underlying these chromosomal rearrangements.

We report a new case of complete trisomy 17p syndrome, which was inherited from a paternal balanced translocation t(15;17)(q11.2;q11.2). Combining our results of the current case with previously reported literature, we further review the relationship between duplication regions and phenotypes.

## 2 MATERIALS AND METHODS

This study was approved by the Ethics Committee of the First Hospital of Jilin University (No. 2020‐378). Written informed consent was obtained from the patient for publication of this case report.

### 2.1 Cytogenetic analysis

Amniotic fluid was collected by amniocentesis at 24 weeks of gestation. Fetal cells were collected by 15‐mL centrifuge tubes for culture. Peripheral blood of the parents was collected by a standard vacuum extraction blood collecting system, which contained EDTA and heparin. Routine sample preparation and chromosome analysis were performed using G‐banding techniques at a resolution of 300‐400 banding in accordance with laboratory standard protocols. Twenty metaphases were counted and at least six karyotypes were analyzed. The karyotypes were described according to the International System for Human Cytogenetic Nomenclature (ISCN 2016).

### 2.2 Single nucleotide polymorphism array

By using the QIAamp DNA MINI kit (Qiagen), genomic DNA was extracted from 10 mL of uncultured amniotic fluid cells in accordance with the manufacturer's instructions. Single nucleotide polymorphism (SNP) array analysis was performed using the Human CytoScan 750K BeadChip (Affymetrix). Thresholds for genome‐wide screening were set at ≥200 kb for gains and ≥100 kb for losses. The quality control standards of copy number variation calling included the following: (a) DNA concentrations ≥50 ng/μL; (b) purified PCR products ≥300 ng/μL, with an OD 260/280 of approximately 1.9 and an OD 260/230 of approximately 2.0; (c) fragmentation products on 1% gel electrophoresis were 25‐125 bp; and (d) MAPD ≤ 0.25, SNPQC ≥ 15.0, and waviness SD ≤ 0.12.

The clinical significance of copy number variation results was determined using open‐access databases. These databases included the (a) Database of Genomic Variants (http://dgv.tcag.ca/dgv/app/home), (b) Database of Chromosomal Imbalance and Phenotype in Human using Ensembl Resources (http://decipher.sanger.ac.uk/), (c) International Standards for Cytogenomic Arrays Consortium Database (https://www.iscaconsortium.org/), (d) European Cytogeneticists Association Register of Unbalanced Chromosome Aberrations (http://www.ecaruca.net), (e) Online Mendelian Inheritance in Man (OMIM) (http://www.ncbi.nlm.nih.gov/omim), and (f) the National Center for Biotechnology Information (NCBI).

## 3 CASE REPORT

**Figure 1:**
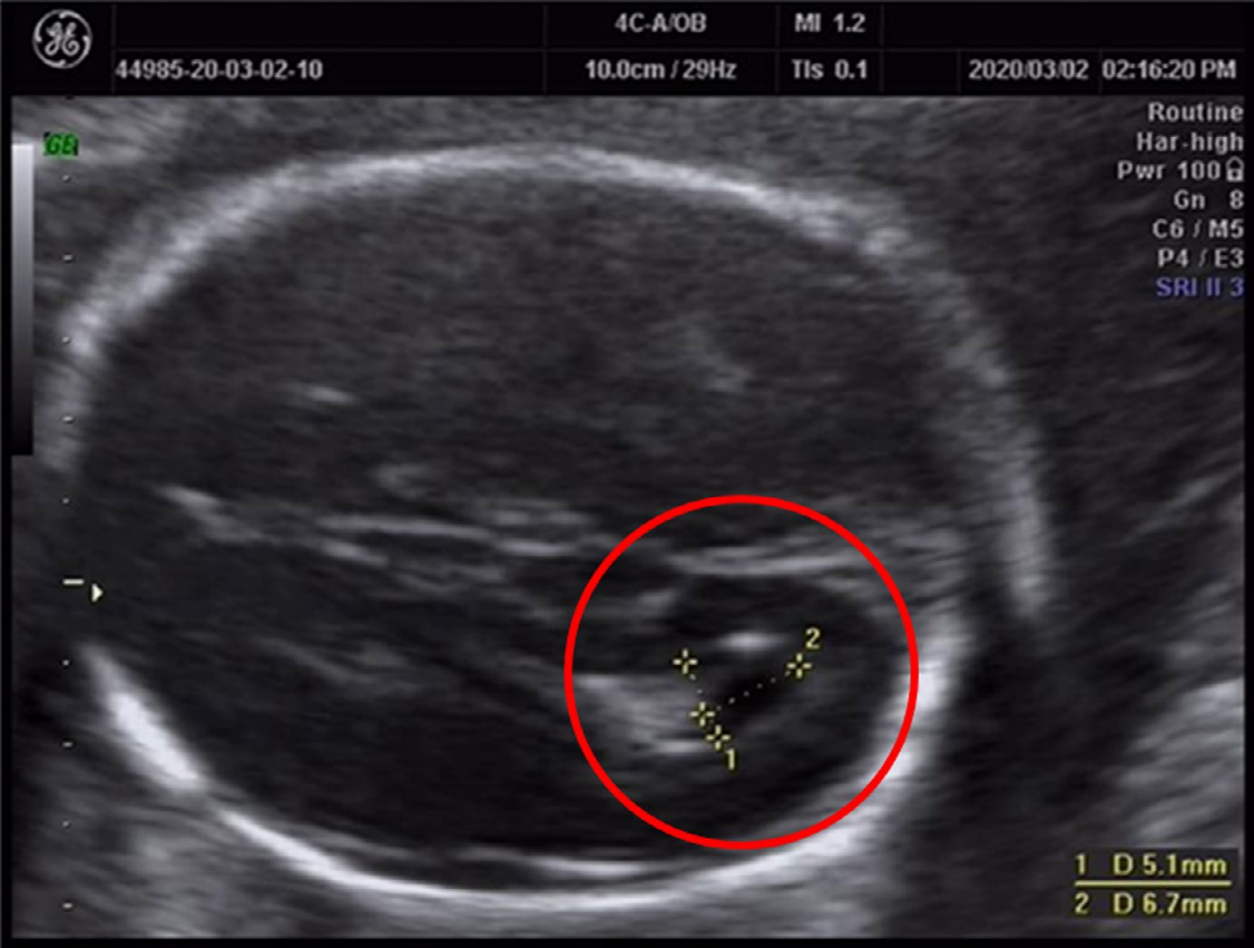
Prenatal ultrasound at 24 wks of gestation shows that the fetus has a right choroid plexus cyst

A 29‐year‐old, gravida 1, para 0, pregnant woman underwent an ultrasonographic examination at 24 weeks of gestation. Ultrasound showed that the biparietal diameter was 47.1 mm, head circumference was 179.8 mm, abdominal circumference was 148.3 mm, femur length was 32.3 mm, and humerus length was 31.6 mm. These indices suggested that the gestational age was 20 weeks, which indicated intrauterine growth retardation. Ultrasonographic evaluation also showed a right choroid plexus cyst (Figure 1), and the gallbladder was not observed. Fetal echocardiography showed a small amount of tricuspid regurgitation, and other indices were normal. After genetic counseling, amniocentesis was performed, and amniotic fluid was collected for cytogenetic and SNP array analyses. Peripheral blood of the parents was collected for cytogenetic analysis.

**Figure 2:**
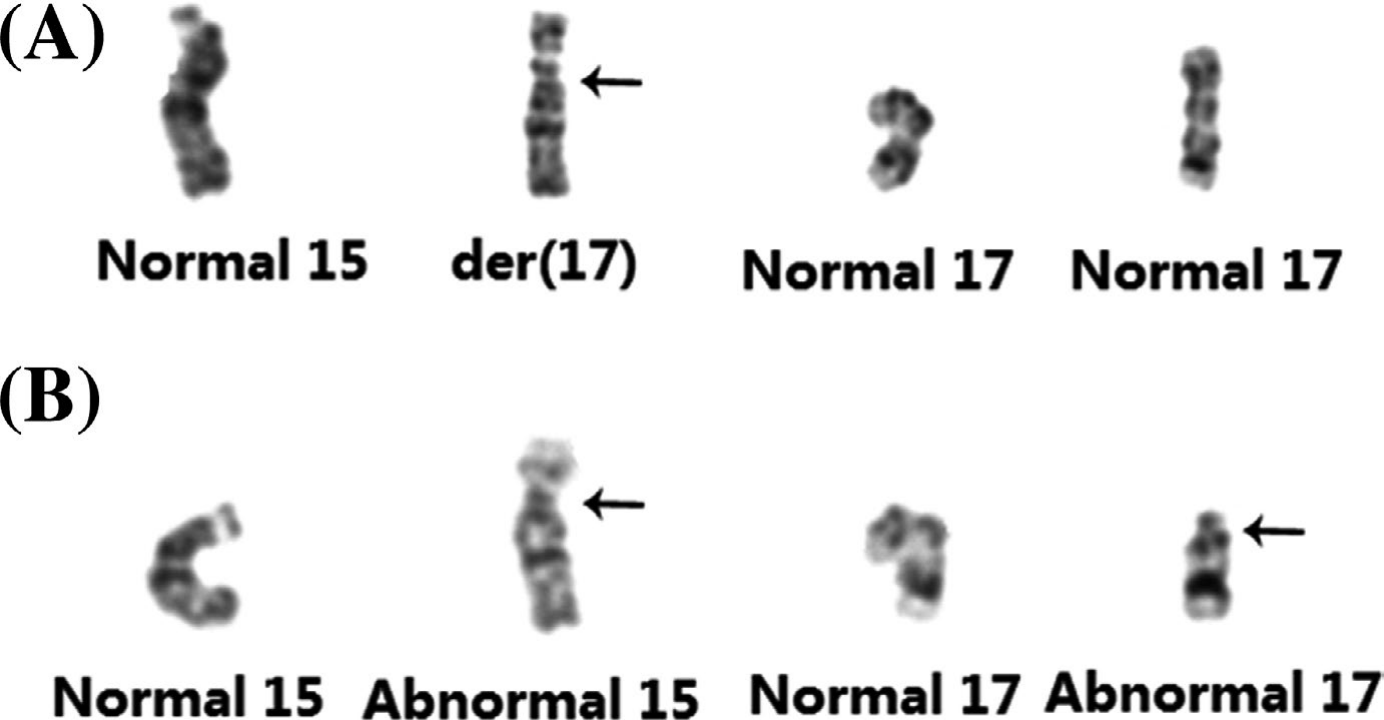
G‐banded chromosomes 15 and 17 from the fetus (A) and the father (B)

**Figure 3:**
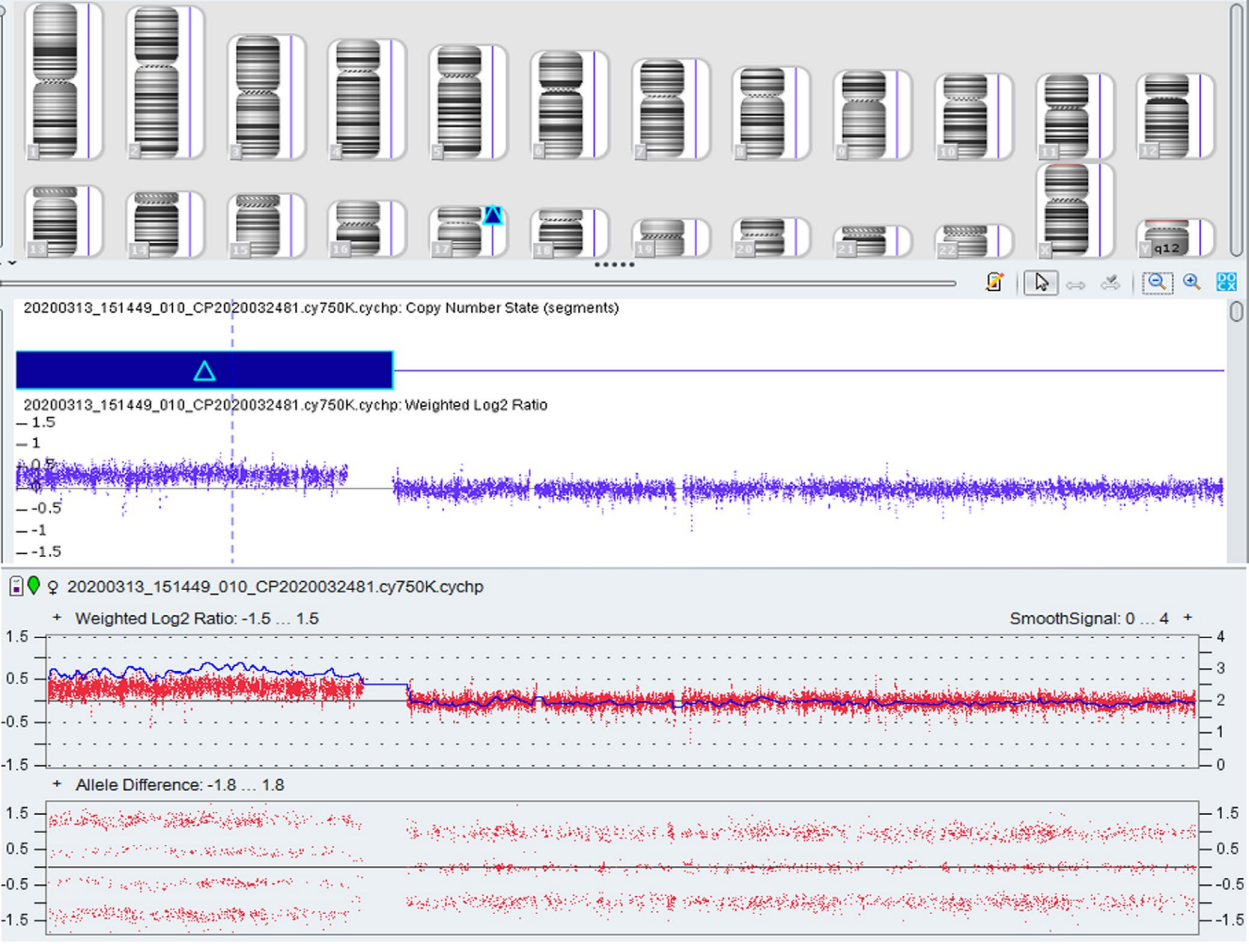
Single nucleotide polymorphism array on uncultured amniocytes shows a 25.309‐Mb duplication in 17p13.3q11.1

The fetal karyotype was 46,XX,der(17)t(15;17)(q11.2;q11.2)pat (Figure 2A). The father's karyotype was 46,XY,t(15;17)(q11.2;q11.2) (Figure 2B), and the mother's karyotype was 46,XX. The SNP array showed arr[GRCh37] 17p13.3q11.1(525‐25309337) × 3, which indicated a 25.309‐Mb duplication in 17p13.3q11.1 (Figure 3). After genetic counseling and informed consent, the parents opted to terminate the pregnancy.

## 4 DISCUSSION

We report a case of complete trisomy 17p syndrome. The abnormal chromosome of the fetus was inherited from its father who had chromosome t(15;17)(q11.2;q11.2). SNP array detection showed that the additional segment on the short arm of chromosome 15 was 17q11.2‐pter. Therefore, we speculated that the fetal karyotype was 46,XX,der(17)t(15;17)(q11.2;q11.2)pat, which belongs to complete trisomy 17p syndrome.

Human chromosome 17 is well known by its duplication structure and complex rearrangement.^9^ Several frequent, unbalanced rearrangements are associated with 17p, and these contain some region‐specific low‐copy repeat sequences. Structural rearrangement of low‐copy repeat sequences in 17p leads to genomic disorders.^10^, ^11^, ^12^ Potocki‐Lupski syndrome is a neurodevelopmental disorder caused by a duplication. This syndrome is mapped within chromosome 17p11.2 and encompasses retinoc acid‐induced gene 1 (*RAI1*). The clinical medical manifestations of this syndrome include hypotonia, congenital heart disease, oropharyngeal dysphagia leading to failure to thrive, mildly dysmorphic facial features, and hypoglycemia.^13^ Charcot‐Marie‐Tooth disease is caused by a duplication of 17p12 containing the peripheral myelin protein 22 (*PMP22*) gene. The typical phenotypes of Charcot‐Marie‐Tooth disease are scoliosis, restless legs syndrome, hip dysplasia, hearing loss, and tremors.^14^ The segment of 17p13.3 includes the tyrosine 3‐monooxygenase/tryptophan 5‐monooxygenase activation protein, epsilon polypeptide (*YWHAE*), and platelet‐activating factor acetylhydrolase isoform 1b regulatory subunit 1 (*PAFAH1B1*) genes. The *YWHAE* gene is closely related to autism, developmental delay, learning delay, and other disorders. The *PAFAH1B1* gene is associated with developmental delay, microcephaly, small body size, and other brain malformations.^15^ On the basis of these genotype‐phenotype relationships, the clinical manifestation of complete trisomy 17p may be more complex than originally thought.

Partial or complete trisomy of chromosome 17p is relatively rare. The first report of trisomy 17p was described by Latta et al in 1974.^16^ Since that time, more cases have been reported as described below. Even though the breakpoint of the duplication segment varies, the phenotype has a recognizable clinical pattern with some common features. These features include prenatal and postnatal growth retardation, psychomotor delay, short webbed neck, microcephaly, micrognathia, high arched palate, ptosis, hypotonia, cardiac malformations, malformed ears, and abnormality of the iris.^3^, ^4^, ^5^, ^17^, ^18^ Most cases of trisomy 17p were described in newborns and childhood. A few cases were diagnosed by three‐dimensional ultrasound in the prenatal period.^3^, ^5^, ^18^, ^19^ Clinical phenotypic characteristics of trisomy 17p according to a review of the literature are shown in Table 1. For cases of different types for trisomy 17p, some of the clinical phenotypic characteristics are consistent, and these include prenatal growth retardation, microcephaly, congenital heart disease, hypotonia, and clinodactyly/hand deformities. In six cases with unbalanced translocation in Table 1, none of the clinical phenotypic characteristics were completely consistent. Therefore, further study of more cases is required.

**Table 1:** Clinical phenotypic characteristics with trisomy of the short arm of chromosome 7 in our case and in the published literature

Reference	Age at diagnosis	Abnormal types	Sex	Breakpoint site	Clinical phenotypic characteristics
Park et al^6^	9 Y	Inv(17)	M	17p11.2 → p13.3	Prenatal growth retardation, microcephaly, low‐set ear, congenital heart disease, hypotonia, flexion deformities, clinodactyly/hand deformities, wide spaced nipples, abnormalities of the iris, biliary atresia
Paskulin et al^2^	Newborn	Der(17)	F	17p11.2 → pter	Prenatal growth retardation, microcephaly, micrognathia, short webbed neck, congenital heart disease, hypotonia, clinodactyly/hand deformities, broad nasal base/bridge, abnormalities of the iris
Latta et al^16^	Newborn	Extra marker	F	17p11 → pter	Prenatal growth retardation(2/2), microcephaly(2/2), hypertelorism(2/2), low‐set ear(2/2), micrognathia(2/2), cleft lip/plate(1/2), short webbed neck(2/2), congenital heart disease(1/2) hypotonia(2/2), flexion deformities (1/2), clinodactyly/hand deformities(2/2), wide spaced nipples(2/2), hydrocephalus (1/2)
Kulharya et al^3^	Newborn		M	17p11.2 → pter	
De Pater et al^19^	Newborn	Unbalanced translocation	F	17p11 → pter	
Lurie et al^18^	22 wks of preg.		F	17p11.2 → pter	Prenatal growth retardation(4/6), microcephaly(2/6), hypertelorism(3/6), low‐set ear(4/6), micrognathia(3/6), short webbed neck(3/6), congenital heart disease(2/6), hypotonia(2/6), flexion deformities(2/6), clinodactyly/hand deformities(2/6), wide spaced nipples(3/6), hydrocephalus(1/6), broad nasal base/bridge(3/6), hearing loss(1/6), biliary atresia (1/6)
Horváth et al^5^	preg.		M	17p11.2 → pter	
Mikhail et al^1^	8 Y		F	17p11.2 → pter	
Spinner et al^4^	Newborn		F	17p11.2 → pter	
The present case	24 wks of preg.		F	17p11.2 → pter	

Abbreviations: F, female; M, male.

In the present case, ultrasonography showed intrauterine growth retardation, which is detected in 80% of prenatal cases. Ultrasonography also showed a right choroid plexus cyst, but the gallbladder was not observed. The latter finding suggested that the fetus may have had biliary atresia, which has not been mentioned in other reports. Fetal echocardiography showed no obvious abnormalities. Other typical malformations were not found in ultrasonographic findings. This lack of findings may be related to the limitations of ultrasonography in the second trimester. Unfortunately, we were unable to provide the results of the fetal autopsy to compare with ultrasonographic findings because the parents refused consent for this research.

## 5 CONCLUSION

Complete trisomy 17p syndrome has severe malformations. Intrauterine growth retardation is the most typical manifestation of trisomy 17p as shown by ultrasonography in the second trimester of pregnancy. According to the current literature, the genotype‐phenotype relationships of complete trisomy 17p syndrome are not completely consistent. To further delineate these relationships, additional cases are required to provide more information by ultrasonographic findings during pregnancy.
